# Improving Respect and Dignity of Older Lesbian Adults: Interprofessional Approaches for Specialized Care

**DOI:** 10.1093/geroni/igaa057.2069

**Published:** 2020-12-16

**Authors:** Noell Rowan, Stephanie Smith, Tamatha Arms, Kris Hohn

**Affiliations:** University of North Carolina Wilmington, Wilmington, North Carolina, United States

## Abstract

Interprofessional research pertaining to LGBTQ older adult cultural sensitivity training for social workers and nurses is often missing in the empirical literature. Members of the LGBTQ communities become increasingly vulnerable to health disparities as they age and treating clients with respect and dignity is at the forefront of this study. Students and faculty engaged in an interprofessional simulation project with older members of the LGBTQ community to learn health knowledge and applied assessment and brief intervention skills. Quantitative findings (N=58; 23 social work; 35 nursing) indicated increased student health knowledge. Reflection and qualitative findings are included with four primary themes: (a) bias of health care providers, (b) access to quality care, (c) specific health care needs, and (d) health risks of LGBTQ older adults. Specific emphasis is given to reflection and insight of the older lesbian participants about access to care, recognition of significant relationships, and marriage equality.

